# Effectiveness of Internet- and mobile-based psychological interventions for the prevention of mental disorders: a systematic review and meta-analysis protocol

**DOI:** 10.1186/s13643-016-0209-5

**Published:** 2016-02-16

**Authors:** Lasse Sander, Leonie Rausch, Harald Baumeister

**Affiliations:** Department of Rehabilitation Psychology and Psychotherapy, Institute of Psychology, University of Freiburg, Engelbergerstr. 41, Freiburg im Breisgau, D-79085 Germany; Department of Medical Psychology and Medical Sociology, Faculty of Medicine, University of Freiburg, Hebelstr. 29, Freiburg im Breisgau, D-79104 Germany; Department of Clinical Psychology and Psychotherapy, Institute of Psychology and Education, University of Ulm, Albert-Einstein-Allee 47, Ulm, D-89081 Germany

**Keywords:** Prevention, Systematic review, Mental disorders, Internet and mobile-based, Online

## Abstract

**Background:**

Despite progress in developing technologies for health promotion and disease treatment, mental disorders remain highly prevalent. In light of the associated personal and societal burden, primary prevention of new onset disorder can be seen as a primary challenge for health care systems and society. Internet- and mobile-based interventions (IMIs) are a promising approach to scale up preventive measures to a population level. The present protocol describes the rationale and design of a systematic review on the effectiveness of IMIs for the prevention of mental disorders.

**Methods/design:**

A systematic literature search (MEDLINE, PsycINFO, CENTRAL) will be conducted. Keywords will target the topics “prevention”, “mental disorders”, and “Internet and mobile-based interventions”. Studies eligible for inclusion will be retrieved, and data will be extracted and evaluated (design, population, outcomes, sample size, duration of intervention and follow-up, drop-out rate) by two independent researchers. Quality of evidence will be assessed, and results will be synthesized qualitatively and pooled meta-analytically when outcome data are comparable in terms of endpoints, assessments, and target disorders.

**Discussion:**

Prevention of mental disorders is one of the major emerging global health challenges. This review and meta-analysis will contribute to our understanding of the potential role for IMIs to help address this challenge.

**Systematic review registration:**

PROSPERO CRD42015026781

**Electronic supplementary material:**

The online version of this article (doi:10.1186/s13643-016-0209-5) contains supplementary material, which is available to authorized users.

## Background

Mental disorders are highly prevalent [1] and associated with a high disease burden for affected individuals and society [2–4]. Wittchen and colleagues reported a 1-year prevalence rate of mental disorders (27 diagnoses included) in the EU population of 38.2 %. Mental disorders are prevalent across age groups, with anxiety disorders (14.0 %), insomnia (7.0 %), and major depression (6.9 %) being the most frequent disorders [1]. Compared to 2005 data, these authors note that neither a substantial decrease in the prevalence nor improvements in care or treatment were found, and less than a third of people affected received any treatment [5]. Thus, despite increasing effectiveness levels of available treatments and utilizing them more widely, interventions to reduce the rate of onset of mental disorder must also be developed and implemented into health care systems. Experts agree that prevention of mental disorders, when possible, reduces burden most significantly [6]. Efforts to support the development of effective preventive interventions are urgently needed.

Recent reviews and meta-analyses highlight that psychological preventive interventions are able to substantially reduce rates of new onset of different types of mental disorders [7–10]. Not surprisingly, studies examining the effects of preventive measures aimed at populations carrying potential risk indicators (=selective prevention) or populations with prodromal syndromes or biological markers to mental disorders (=indicated prevention) are more common than studies geared toward the general population, regardless of risk status (=universal prevention) [7].

Using the Internet as the medium for the delivery of psychological prevention interventions has several advantages compared to traditional intervention settings. Internet- and mobile-based interventions (IMIs) are easy to access, enabling users to flexibly integrate interventions into their daily life without local or temporal boundaries [11]. Moreover, users can set the pace for working with these interventions and can go through each session as frequently or as quickly as they like. For users fearing stigmatization, the anonymity offered by IMIs can be appealing. First studies also found Internet-based interventions to be cost-effective [12–14], a crucial element in light of limited resources in many health care systems.

Additionally, access to and use of the Internet have grown rapidly over the last decade across the globe [15]. Mental health professionals could leverage this trend of increasing Internet use, which often includes getting health information and support [16].

Several studies have examined the effectiveness of Internet-based prevention interventions for specific mental disorders (e.g., [17, 18]). Prior reviews and meta-analyses have been published focusing on IMIs for the prevention of eating disorders [19–21] and substance-related/addictive disorders [22, 23]. To our knowledge, there is no previous systematic literature review summarizing the existing research on Internet- and mobile-based prevention interventions for mental disorders in general. Therefore, the results of this review and meta-analysis will provide an overview of this field of research that can benefit clinicians, public health policy makers, and researchers. The present protocol describes the rationale and design of the systematic review and meta-analysis.

## Methods/design

This review is registered with PROSPERO (registration CRD42015026781).

### Aims

The review aims to synthesize the evidence base of randomized controlled trials (RCTs) reporting the effectiveness of Internet- and mobile-based prevention interventions of mental disorders. The review will be reported according to the Preferred Reporting Items for Systematic Reviews and Meta-Analyses (PRISMA) guidelines [24].

### Search strategy

Relevant articles will be identified by searching electronic databases. Databases included are Cochrane Central Register of Controlled trials (CENTRAL), PsycINFO, and MEDLINE. A sensitive search strategy will be used (see Additional file 1). The search will be complemented by a review of reference lists from identified publications and a search of the WHO International Clinical Trials Registry Platform (ICTRP) to include ongoing trials. When indicated, study authors will be contacted to obtain further information in order to clarify study characteristics. When study protocols are identified without subsequent publication of results, authors will be contacted to obtain missing or unpublished data and determine eligibility for inclusion in this review.

### Eligibility criteria

Population: Studies are eligible for inclusion if they (a) focus on an adult target population and (b) include adults without a diagnosis of the target mental disorder at baseline (primary prevention intervention). (c) Mental disorder should be assessed by means of standardized interviews (e.g., SCID, CIDI), validated self-reports (e.g., BDI), clinician-rated scales (e.g., HAMD) with normed cut-off points, or diagnosis by health care professionals. Studies on the prevention of substance-related/addictive disorders will be excluded, as this represents a frequently studied and already elsewhere reviewed specific subgroup of prevention research [22, 23] and these interventions typically follow an educational or health behavior change model rather than a psychotherapeutic intervention model (see (d)).

Intervention: (d) Interventions must be based on psychological interventions. The definition of “psychological intervention” is taken from Kampling and colleagues [25] and refers to cognitive behavioral therapy, psychodynamic psychotherapy, behavior therapy or behavior modification, systemic therapy, third wave cognitive behavioral therapies, humanistic therapies, integrative therapies, and other psychological-oriented interventions. (e) Interventions must be provided in an online setting, defined as online-, Internet-, Web-, or mobile-based. Interventions may vary concerning the amount of external guidance provided to participants. Self-help interventions will also be included. We will exclude studies on the relapse prevention of mental disorder, as these treatment maintenance interventions differ substantially from preventive interventions focused on the first or recurrent onset of mental disorders [25].

Comparison: (f) Studies must include a control group. This can be (enhanced) usual care, wait-list control group, another intervention, or no treatment.

Outcomes: (g) Studies examining onset will be considered*,* defined as the percentage of persons who developed the mental disorder under study from pre- to follow-up assessment. In addition to the data from standardized clinical interviews (e.g., SCID-IV [26]), we will include studies reporting only reporting symptom severity scores, when validated rating scales with normed cut-off points (referencing onset of disorder or diagnosis) have been used. To be able to meaningfully comment on any post-intervention reduction of incidence, studies must (h) include a follow-up assessment at 3 months or longer post-randomization.

Study type: (i) only RCTs that are available in full text will be eligible for this review. Studies must be published in English or German.

### Study selection process

**Fig. 1:**
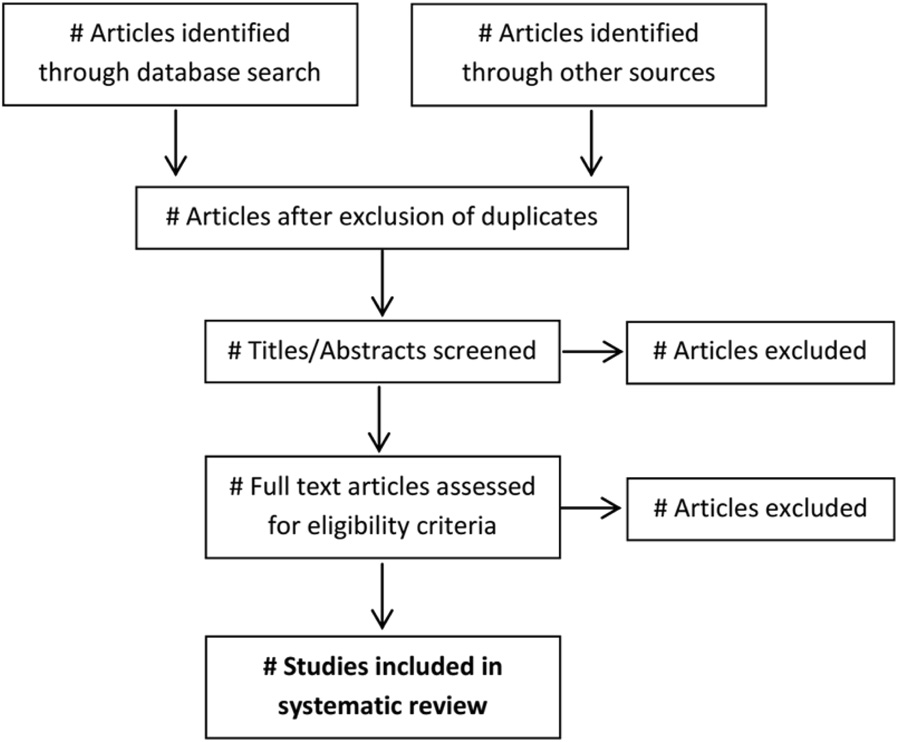
Study selection process (adapted from [24])

The selection of articles will be conducted by two independent reviewers (LS, LR). In the first step, they will screen all titles and abstracts yielded by the database search. In the second step, full texts of the selected articles will be retrieved and screened in terms of the aforementioned eligibility criteria. Reference lists of finally included articles will be screened in the same way. Disagreement will be resolved by a discussion among the reviewers. When needed to resolve disagreement, a third reviewer (HB) will be consulted. To illustrate the study selection process and reasons for exclusion, a PRISMA flow chart [24] will be provided (see Fig. 1).

### Data extraction

The following data items will be extracted for each study, when available: (a) study identification items (first author, year of publication), (b) study design characteristics (sample size, intervention design/type, level of human support/guidance target mental disorder, control group, type of assessments, duration of intervention, length of follow-up assessments), (c) target population items (age, gender), (d) setting (nationality, environment, recruitment strategy), (e) drop-out rate and (f) clinical outcomes (onset and/or severity of disorder). If outcomes are assessed by several instruments, data will be extracted as follows: (1) Onset data from structured interviews will be prioritized. (2) Data from clinician-rated scales will be prioritized over self-report questionnaires. (3) Self-report questionnaires will be prioritized over diagnosis by health care professionals. (4) In case where several outcome measures of the same hierarchy level are used in one study, we will select the outcome measure that is used most frequently across eligible studies or else randomly select one outcome measure. To avoid bias, a second reviewer will check the extracted data.

### Quality assessment

In order to evaluate the quality of research, two independent reviewers will assess the risk of bias using the Cochrane Collaboration’s tool for assessing risk of bias in RCTs [27]. As recommended, each study will be assessed for procedures in the following domains: (a) random sequence generation, (b) allocation concealment, (c) blinding of participants, personnel, and outcome assessors, (d) incomplete outcome data (drop-out, intention-to-treat), (e) selective outcome reporting, and (f) other threats to validity. Studies will be rated as showing a “low” or “high” risk of bias according to the aforementioned criteria. Of note, in the implementation of psychological interventions, blinding of health care providers (if a guided intervention was provided) or patients concerning the treatment is not possible. This results in a “high” risk of bias rating on this criterion. However, outcome assessors can remain unaware of the treatment allocation of patients.

#### Data synthesis and presentation

A detailed description of the results for all included studies will be provided in text and tables. Characteristics of selected studies will be listed and qualitatively described. Characteristics include study design and characteristics (sample size, duration, follow-up period), patient population (age, gender), intervention characteristics (name, intervention content (e.g., CBT), technical implementation (e.g., Web-only or Web- and mobile-based), duration, level of human support/guidance), study and intervention drop-out rate, assessment tool used to determine presence of mental disorder (clinical interview, questionnaire), type of prevention (universal, selective, indicative), recruitment procedure, target disorder to be prevented, and any covariates assessed (list of variables).

### Meta-analysis

Data analysis will be performed using Review Manager 5.3 software from the Cochrane Collaboration. Meta-analytic pooling will be conducted when at least three studies report outcome parameters on the same specific mental disorder (i.e., specific mental disorder according to DSM/ICD such as major depression or social phobia assessed as described in “eligibility criteria: population: (c)”). Only studies showing less than substantial statistical heterogeneity will be pooled. Heterogeneity will be evaluated with the *I*^2^ statistic. An *I*^2^ of 0–60 % can be regarded as not important to moderate, while *I*^2^ > 60 % indicates substantial heterogeneity [27]. Focusing on specific mental disorders rather than pooling results across disorder entities will limit clinical heterogeneity of pooled estimates. The random-effects method using the inverse-variance model for pooled estimates on the prevention of mental disorders (hazard ratios, odds ratios with 95 % CI) and for the pooled standardized mean difference (SMD with 95 % CI) of the severity level of the respective mental disorder will be used. Outcome assessment data (i.e., on the onset and the severity differences between trial arms) will be pooled, subdivided for short-term (post-treatment assessment), medium-term (≤6 months post-randomization follow-up), and long-term (> 6 months post-randomization follow-up) trial effects. For further comparisons concerning content and form of intervention, subgroup analysis will be performed if feasible.

## Discussion

This systematic review and meta-analysis will add to the previous research by summarizing, synthesizing, and discussing the existing literature on Internet- and mobile-based prevention interventions for mental disorders. The findings of this review will extend beyond previous systematic reviews in this field (e.g., [20, 28]), as results will extend to a more comprehensive range of mental disorders. Thus, the proposed systematic review and meta-analysis will provide a valuable overview and synthesis of the entire field of Internet- and mobile-based mental disorder prevention interventions for clinical practitioners, researchers, and public health policy makers.

The following characteristics will contextualize the findings and generalizability of this review. In service of providing a broad review on the broad scope of mental disorders, this review will include trials that might differ substantially regarding their target population, methods, interventions, control groups, assessments, and outcomes. While this will provide a comprehensive overview on the prevention of mental disorders by means of Internet- and mobile-based interventions, heterogeneity in clinical, methodological, and statistical approaches will necessarily limit the amount of quantitative pooling of studies. Moreover, the language bias (including only studies published in English or German) may lead to an overestimation of effects, as statistically significant results are more likely to be published in the English language [29]. The publication bias of only significant findings being published may also contribute to the limitation of results [30]. To address this issue, we will attempt to include unpublished and non-significant studies by contacting principal investigators of studies and study protocols.

In the past, efforts toward prevention of mental disorders were often limited due to limited resources of health care systems as well as challenges inherent in providing an intervention en masse [31]. Using the Internet as the medium for provision has the potential to overcome this limitation. To guide public health policy, the level of effectiveness of an intervention is a crucial information for decisions on the implementation of new health care approaches. An important issue concerning Internet- and mobile-based interventions is attrition [32–34]. We will therefore list and discuss treatment and intervention drop-out rates in this review.

The proposed review will go beyond summarizing the existing evidence by also qualitatively analyzing the past and ongoing interventions. In this way, gaps in the previous research will be addressed, essential characteristics of prevention interventions will be outlined, and missing subfields in research will be identified.

Given the numerous upcoming Internet- and mobile-based prevention intervention trials on mental disorders and the absence of a respective systematic review, the proposed review is urgently needed and will substantially add to the current evidence. Our findings will help researchers to identify options for future directions and public health policy makers to estimate the potential of mental health prevention interventions.

## Additional file

Additional file 1:
**Table showing the search strings for CENTRAL, MEDLINE, and PsycINFO.**

## Abbreviations

CENTRAL: Cochrane Central Register of Controlled Trials
ICTRP: International Clinical Trials Registry Platform
IMI: Internet and mobile-based intervention
PRISMA: Preferred Reporting Items for Systematic Reviews and Meta-Analyses
RCT: randomized controlled trial
WHO: World Health Organization
