# Erratum to: Effectiveness of Internet- and mobile-based psychological interventions for the prevention of mental disorders: a systematic review and meta-analysis protocol

**DOI:** 10.1186/s13643-016-0332-3

**Published:** 2016-09-14

**Authors:** Lasse Sander, Leonie Rausch, Harald Baumeister

**Affiliations:** 1 Department of Rehabilitation Psychology and Psychotherapy, Institute of Psychology, University of Freiburg, Engelbergerstr. 41, Freiburg im Breisgau, D-79085 Germany; 2 Department of Medical Psychology and Medical Sociology,Faculty of Medicine, University of Freiburg, Hebelstr. 29, Freiburg im Breisgau, D-79104 Germany; 3 Department of Clinical Psychology and Psychotherapy, Institute of Psychology and Education, University of Ulm, Albert-Einstein-Allee 47, Ulm, D-89081 Germany

## Erratum

After publication of the original article [1], it was noticed that the ORCID of Lasse Sander was accidentally omitted. This is now included in this erratum: “orcid.org/0000-0002-4222-9837”.
