# Supplementary material for: Effectiveness of Internet- and mobile-based psychological interventions for the prevention of mental disorders: a systematic review and meta-analysis protocol
Source: Syst Rev. 2016 Feb 16;5:30. doi: 10.1186/s13643-016-0209-5 (PMC4754873; doi:10.1186/s13643-016-0209-5)
Supplement: Additional file 1: — Table showing the search strings for CENTRAL, MEDLINE, and PsycINFO. [file 13643_2016_209_MOESM1_ESM.pdf]

Additional File 1. Table showing the search strings for CENTRAL, MEDLINE and PsycINFO

	CENTRAL	MEDLINE	PsycINFO
S1	MeSH descriptor: [Telemedicine] explode all trees	(MM "Telemedicine+")	MM "Telemedicine"
S2	MeSH descriptor: [Therapy, Computer-Assisted] explode all trees	(MM "Therapy, Computer-Assisted+")	MM "Computer Assisted Therapy"
S3	MeSH descriptor: [Computer-Assisted Instruction] explode all trees	(MM "Computer-Assisted Instruction")	MM "Computer Assisted Instruction+"
S4	MeSH descriptor: [Mobile Applications] explode all trees	(MM "Mobile Applications")	MM "Mobile Devices+"
S5	MeSH descriptor: [Internet] explode all trees	(MM "Internet+")	MM "Internet"
S6	"computer applications": ti,ab,kw in Trials	AB "computer applications"	AB "computer applications"
S7	"online therapy":ti,ab,kw in Trials	AB "online therapy"	AB "online therapy"
S8	online:ti,ab,kw in Trials	AB online	AB online
S9	mobile:ti,ab,kw in Trials	AB mobile	AB mobile
S10	eHealth:ti,ab,kw in Trials	AB eHealth	AB eHealth
S11	web-based:ti,ab,kw in Trials	AB web-based	AB web-based
S12	computer-based:ti,ab,kw in Trials	AB computer-based	AB computer-based
S13	computerized:ti,ab,kw in Trials	AB computerized	AB computerized
S14	"world wide web":ti,ab,kw in Trials	AB "world wide web"	AB "world wide web"
S15	cyber:ti,ab,kw in Trials	AB cyber	AB cyber
S16	#1 or #2 or #3 or #4 or #5 or #6 or #7 or #8 or #9 or #10 or #11 or #12 or #13 or #14 or #15	S1 OR S2 OR S3 OR S4 OR S5 OR S6 OR S7 OR S8 OR S9 OR S10 OR<br>S11 OR S12 OR S13 OR S14 OR S15	S1 OR S2 OR S3 OR S4 OR S5 OR S6 OR S7 OR S8 OR S9 OR S10 OR<br>S11 OR S12 OR S13 OR S14 OR S15
S17	MeSH descriptor: [Mental Disorders] explode all trees	(MM "Mental Disorders+")	MM "Mental Disorders+"
S18	MeSH descriptor: [Mentally Ill Persons] explode all trees	(MM "Mentally Ill Persons")	AB "mentally ill persons"
S19	"mental distress":ti,ab,kw in Trials	AB "mental distress"	AB "mental distress"
S20	"psychiatric disorder*":ti,ab,kw in Trials	AB "psychiatric disorder*"	AB "psychiatric disorder*"
S21	"psychological disorder*": ti,ab,kw in Trials	AB "psychological disorder*"	AB "psychological disorder*"
S22	"mental illness*":ti,ab,kw in Trials	AB "mental illness*"	AB "mental illness*"
S23	MeSH descriptor: [Substance-Related Disorders] explode all trees	(MM "Substance-Related Disorders+")	AB "substance-related disorder*"
S24	MeSH descriptor: [Alcohol-Related Disorders] explode all trees	(MM "Alcohol-Related Disorders+")	AB "alcohol-related disorder*"
S25	"alcohol dependence":ti,ab,kw in Trials	AB "alcohol dependence"	AB "alcohol dependence"
S26	"alcohol abuse":ti,ab,kw in Trials	AB "alcohol abuse"	AB "alcohol abuse"
S27	"substance abuse":ti,ab,kw in Trials	AB "substance abuse"	AB "substance abuse"

Additional File 1. Table showing the search strings for CENTRAL, MEDLINE and PsycINFO

S28	MeSH descriptor: [Psychotic Disorders] explode all trees	(MM "Psychotic Disorders+")	AB "psychotic disorder*"
S29	MeSH descriptor: [Schizophrenia] explode all trees	(MM "Schizophrenia+")	MM "Schizophrenia+"
S30	MeSH descriptor: [Affective Disorders, Psychotic] explode all trees	(MM "Affective Disorders, Psychotic+")	AB "psychotic affective disorder*"
S31	MeSH descriptor: [Mood Disorders] explode all trees	(MM "Mood Disorders+")	AB "mood disorder*"
S32	MeSH descriptor: [Depressive Disorder, Major] explode all trees	(MM "Depressive Disorder, Major")	MM "Major Depression+"
S33	MeSH descriptor: [Depression] explode all trees	(MM "Depression")	MM "Recurrent Depression"
S34	MeSH descriptor: [Bipolar Disorder] explode all trees	(MM "Bipolar Disorder+")	MM "Bipolar Disorder+"
S35	MeSH descriptor: [Dysthymic Disorder] explode all trees	(MM "Dysthymic Disorder")	MM "Dysthymic Disorder"
S36	MeSH descriptor: [Depressive Disorder] explode all trees	(MM "Depressive Disorder+")	AB "depressive disorder*"
S37	MeSH descriptor: [Seasonal Affective Disorder] explode all trees	(MM "Seasonal Affective Disorder")	MM "Seasonal Affective Disorder"
S38	"affective disorder*":ti,ab,kw in Trials	AB "affective disorder*"	MM "Affective Disorders+"
S39	depressive:ti,ab,kw in Trials	AB depressive	AB depressive
S40	depression:ti,ab,kw in Trials	AB depression	AB depression
S41	MeSH descriptor: [Anxiety] explode all trees	(MM "Anxiety+")	MM "Anxiety+"
S42	MeSH descriptor: [Anxiety Disorders] explode all trees	(MM "Anxiety Disorders+")	MM "Anxiety Disorders+"
S43	MeSH descriptor: [Panic] explode all trees	(MM "Panic")	MM "Panic"
S44	MeSH descriptor: [Panic Disorder] explode all trees	(MM "Panic Disorder")	MM "Panic Disorder"
S45	MeSH descriptor: [Phobic Disorders] explode all trees	(MM "Phobic Disorders")	MM "Phobias+"
S46	MeSH descriptor: [Agoraphobia] explode all trees	(MM "Agoraphobia")	AB agoraphobia
S47	"anxiety disorder*":ti,ab,kw in Trials	AB "anxiety disorder*"	AB "anxiety disorder*"
S48	panic:ti,ab,kw in Trials	AB panic	AB panic
S49	phobi*:ti,ab,kw in Trials	AB phobi*	AB phobi*
S50	MeSH descriptor: [Obsessive-Compulsive Disorder] explode all trees	(MM "Obsessive-Compulsive Disorder+")	MM "Obsessive Compulsive Disorder"
S51	MeSH descriptor: [Impulse Control Disorders] explode all trees	(MM "Impulse Control Disorders+")	MM "Impulse Control Disorders+"
S52	MeSH descriptor: [Stress Disorders, Post-Traumatic] explode all trees	(MM "Stress Disorders, Post-Traumatic")	MM "Posttraumatic Stress Disorder"
S53	MeSH descriptor: [Stress Disorders, Traumatic] explode all trees	(MM "Stress Disorders, Traumatic+")	AB "traumatic stress disorder*"

Additional File 1. Table showing the search strings for CENTRAL, MEDLINE and PsycINFO

S54	MeSH descriptor: [Adjustment Disorders] explode all trees	(MM "Adjustment Disorders")	MM "Adjustment Disorders"
S55	PTSD:ti,ab,kw in Trials	AB PTSD	AB PTSD
S56	"posttraumatic stress disorder*":ti,ab,kw in Trials	AB "posttraumatic stress disorder*"	AB "posttraumatic stress disorder*"
S57	MeSH descriptor: [Somatoform Disorders] explode all trees	(MM "Somatoform Disorders+")	MM "Somatoform Disorders+"
S58	"somatic symptom disorder*":ti,ab,kw in Trials	AB "somatic symptom disorder*"	AB "somatic symptom disorder*"
S59	somatoform:ti,ab,kw in Trials	AB somatoform	AB somatoform
S60	MeSH descriptor: [Eating Disorders] explode all trees	(MM "Eating Disorders+")	MM "Eating Disorders+"
S61	MeSH descriptor: [Anorexia] explode all trees	(MM "Anorexia")	-
S62	MeSH descriptor: [Anorexia Nervosa] explode all trees	(MM "Anorexia Nervosa")	MM "Anorexia Nervosa"
S63	MeSH descriptor: [Bulimia] explode all trees	(MM "Bulimia")	MM "Bulimia"
S64	MeSH descriptor: [Bulimia Nervosa] explode all trees	(MM "Bulimia Nervosa")	-
S65	MeSH descriptor: [Binge-Eating Disorder] explode all trees	(MM "Binge-Eating Disorder")	MM "Binge Eating Disorder"
S66	anorexia:ti,ab,kw in Trials	AB anorexia	AB anorexia
S67	bulimia:ti,ab,kw in Trials	AB bulimia	AB bulimia
S68	MeSH descriptor: [Sleep Disorders] explode all trees	(MM "Sleep Disorders+")	MM "Sleep Disorders+"
S69	insomnia:ti,ab,kw in Trials	AB insomnia	AB insomnia
S70	#17 or #18 or #19 or #20 or #21 or #22 or #23 or #24 or #25 or #26 or #27 or #28 or #29 or #30 or #31 or #32 or #33 or #34 or #35 or #36 or #37 or #38 or #39 or #40 or #41 or #42 or #43 or #44 or #45 or #46 or #47 or #48 or #49 or #50 or #51 or #52 or #53 or #54 or #55 or #56 or #57 or #58 or #59 or #60 or #61 or #62 or #63 or #64 or #65 or #66 or #67 or #68 or #69	S17 OR S18 OR S19 OR S20 OR S21 OR S22 OR S23 OR S24 OR S25 OR S26 OR S27 OR S28 OR S29 OR S30 OR S31 OR S32 OR S33 OR S34 OR S35 OR S36 OR S37 OR S38 OR S39 OR S40 OR S41 OR S42 OR S43 OR S44 OR S45 OR S46 OR S47 OR S48 OR S49 OR S50 OR S51 OR S52 OR S53 OR S54 OR S55 OR S56 OR S57 OR S58 OR S59 OR S60 OR S61 OR S62 OR S63 OR S64 OR S65 OR S66 OR S67 OR S68 OR S69	S17 OR S18 OR S19 OR S20 OR S21 OR S22 OR S23 OR S24 OR S25 OR S26 OR S27 OR S28 OR S29 OR S30 OR S31 OR S32 OR S33 OR S34 OR S35 OR S36 OR S37 OR S38 OR S39 OR S40 OR S41 OR S42 OR S43 OR S44 OR S45 OR S46 OR S47 OR S48 OR S49 OR S50 OR S51 OR S52 OR S53 OR S54 OR S55 OR S56 OR S57 OR S58 OR S59 OR S60 OR S62 OR S63 OR S65 OR S66 OR S67 OR S68 OR S69
S71	random* in Trials	TX random*	TX random*
S72	"randomized controlled trial" in Trials	TX "randomized controlled trial"	TX "randomized controlled trial"
S73	"controlled clinical trial" in Trials	TX "controlled clinical trial"	TX "controlled clinical trial"
S74	RCT in Trials	TX RCT	TX RCT
S75	"clinical trial" in Trials	TX "clinical trial"	TX "clinical trial"
S76	#71 or #72 #73 #74 #75	S71 OR S72 OR S73 OR S74 OR S75	S71 OR S72 OR S73 OR S74 OR S75
S77	prevent* in Trials	TX prevent*	TX prevent*
S78	MeSH descriptor: [Numbers Needed To Treat] explode all trees	(MM "Numbers Needed To Treat")	-
S79	"number needed to treat":ti,ab,kw in Trials	AB "number needed to treat"	AB "number needed to treat"

Additional File 1. Table showing the search strings for CENTRAL, MEDLINE and PsycINFO

S80	"number needed to be treated":ti,ab,kw in Trials	AB "number needed to be treated"	AB "number needed to be treated"
S81	"relative risk":ti,ab,kw in Trials	AB "relative risk"	AB "relative risk"
S82	onset:ti,ab,kw in Trials	AB onset	AB onset
S83	incidence:ti,ab,kw in Trials	AB incidence	AB incidence
S84	incident:ti,ab,kw in Trials	AB incident	AB incident
S85	#77 or #78 or #79 or #80 or #81 or #82 or #83 or #84	S77 OR S78 OR S79 OR S80 OR S81 OR S82 OR S83 OR S84	S77 OR S78 OR S79 OR S80 OR S81 OR S82 OR S83 OR S84
S86	#16 and #70 and #76 and #85	S16 AND S70 AND S76 AND S85	S16 AND S70 AND S76 AND S85
